# Expression of the Growth Factor Progranulin in Endothelial Cells Influences Growth and Development of Blood Vessels: A Novel Mouse Model

**DOI:** 10.1371/journal.pone.0064989

**Published:** 2013-05-31

**Authors:** Huishi Toh, Mingju Cao, Eugene Daniels, Andrew Bateman

**Affiliations:** 1 Division of Endocrinology and Metabolism, Department of Medicine, Royal Victoria Hospital, McGill University Health Center, Montreal, Quebec, Canada; 2 Department of Anatomy and Cell Biology, Faculty of Medicine, McGill University, Montreal, Quebec, Canada; Katholieke Universiteit Leuven, Belgium

## Abstract

Progranulin is a secreted glycoprotein that regulates cell proliferation, migration and survival. It has roles in development, tumorigenesis, wound healing, neurodegeneration and inflammation. Endothelia in tumors, wounds and placenta express elevated levels of progranulin. In culture, progranulin activates endothelial proliferation and migration. This suggested that progranulin might regulate angiogenesis. It was, however, unclear how elevated endothelial progranulin levels influence vascular growth *in vivo*. To address this issue, we generated mice with progranulin expression targeted specifically to developing endothelial cells using a *Tie2*–promoter/enhancer construct. Three *Tie2-Grn* mouse lines were generated with varying *Tie2-Grn* copy number, and were called GrnLo, GrnMid, and GrnHi. All three lines showed increased mortality that correlates with *Tie2-Grn* copy number, with greatest mortality and lowest germline transmission in the GrnHi line. Death of the transgenic animals occurred around birth, and continued for three days after birth. Those that survived beyond day 3 survived into adulthood. Transgenic neonates that died showed vascular abnormalities of varying severity. Some exhibited bleeding into body cavities such as the pericardial space. Smaller localized hemorrhages were seen in many organs. Blood vessels were often dilated and thin-walled. To establish the development of these abnormalities, we examined mice at early (E10.5–14.5) and later (E15.5–17.5) developmental phases. Early events during vasculogenesis appear unaffected by *Tie2-Grn* as apparently normal primary vasculature had been established at E10.5. The earliest onset of vascular abnormality was at E15.5, with focal cerebral hemorrhage and enlarged vessels in various organs. Aberrant *Tie2-Grn* positive vessels showed thinning of the basement membrane and reduced investiture with mural cells. We conclude that progranulin promotes exaggerated vessel growth *in vivo*, with subsequent effects in the formation of the mural cell layer and weakening of vessel integrity. These results demonstrate that overexpression of progranulin in endothelial cells influences normal angiogenesis *in vivo*.

## Introduction

Angiogenesis, the formation of new blood vessels from preexisting vessels, is critical whenever tissues undergo extensive remodeling processes, including during normal growth and development, wound healing [1] [2], in the female reproductive cycle [3], mammary gland development during pregnancy and lactation [4] and the expansion of fat mass during obesity [5] [6]. Angiogenesis contributes to many pathologies, including the formation of tumor blood supply [7], and contributes to diabetic complications such as retinopathy and kidney failure [8] [9]. Angiogenesis is regulated by vascular-specific growth factors, notably those of the vascular endothelial growth factor (VEGF) family and the angiopoietins, and by less cell-restricted growth factors including members of the fibroblast growth factor family, the Notch family proteins and proteins in the Eph–signaling family [10]. We have proposed previously that the secreted glycoprotein progranulin may also have a role in vascular development [11].

Progranulin (which is also called granulin-epithelin precursor, PC cell derived growth factor and acrogranin) is a pleiotropic regulatory protein that promotes cellular proliferation, cell migration and survival (reviewed in [12]). It is often overexpressed in cancers and contributes to tumor progression (reviewed in [13], [14]) and stroma formation [15]. Mutation of the gene for progranulin (*Grn*) results in the neurodegenerative disorder, frontotemporal dementia [16] [17]. Progranulin regulates inflammation [18] [19] [20]. It is an inhibitor of the tumor necrosis factor receptors [21], and is a cofactor in the presentation of CpG oligonucleotides to the Toll-9 receptor [22]. Quiescent endothelial cells typically express little progranulin [23], however progranulin expression is induced in activated endothelial cells after tissue wounding [11], and in the developing placenta [24]. Progranulin staining has been reported in endothelial cells in ovarian tumors [25] [26], breast cancer [27], esophageal carcinomas, [28], and in gliomas [29]. Indeed, cumulative survival in patients with glioblastoma was significantly decreased for those with overexpression of progranulin in the tumor vasculature [29]. Progranulin stimulates the expression of the angiogenic growth factor VEGF in breast cancer cell lines [30], and breast cancers with elevated progranulin levels also have higher VEGF levels and microvascular density [27]. Monoclonal antibodies that deplete progranulin levels reduced cancer growth of hepatocellular carcinomas in mice, in part by reducing tumor microvascular density [31] by decreasing progranulin mediated VEGF expression. Progranulin increased capillary size and number when it was added to rodent dermal wounds [11]. In tissue culture, progranulin stimulated proliferation and migration of endothelial cells in a mitogen-activated protein kinase (MAPK) and phosphatidyl-inositol-3-kinase (PI3K)-dependent fashion [11]. It also accelerated tubule formation of primary microvascular endothelial cells growing on Matrigel [11]. Tube-formation is an assay of organized self-association *in vitro* that recapitulates the formation of luminal endothelial structures during angiogenesis *in vivo* [32].

Despite the production of progranulin by endothelial cells in tissues that are actively proliferating and remodeling, including tumors, and the ability of progranulin to stimulate endothelial proliferation and migration in tissue culture, it remains uncertain whether progranulin influences angiogenic processes in the more complex *in vivo* environment. To address this issue, we developed mice that overexpress *GRN* in endothelial cells under the control of the *Tie2* promoter. These transgenic mice exhibited expanded vessel size, and a progressive disruption of vascular integrity that leads to high rates of perinatal mortality. This supports the hypothesis that progranulin influences angiogenic processes *in vivo*.

## Results

### Characterization of the *Tie2-Grn* Founder Transgenic Mice

**Figure 1:**
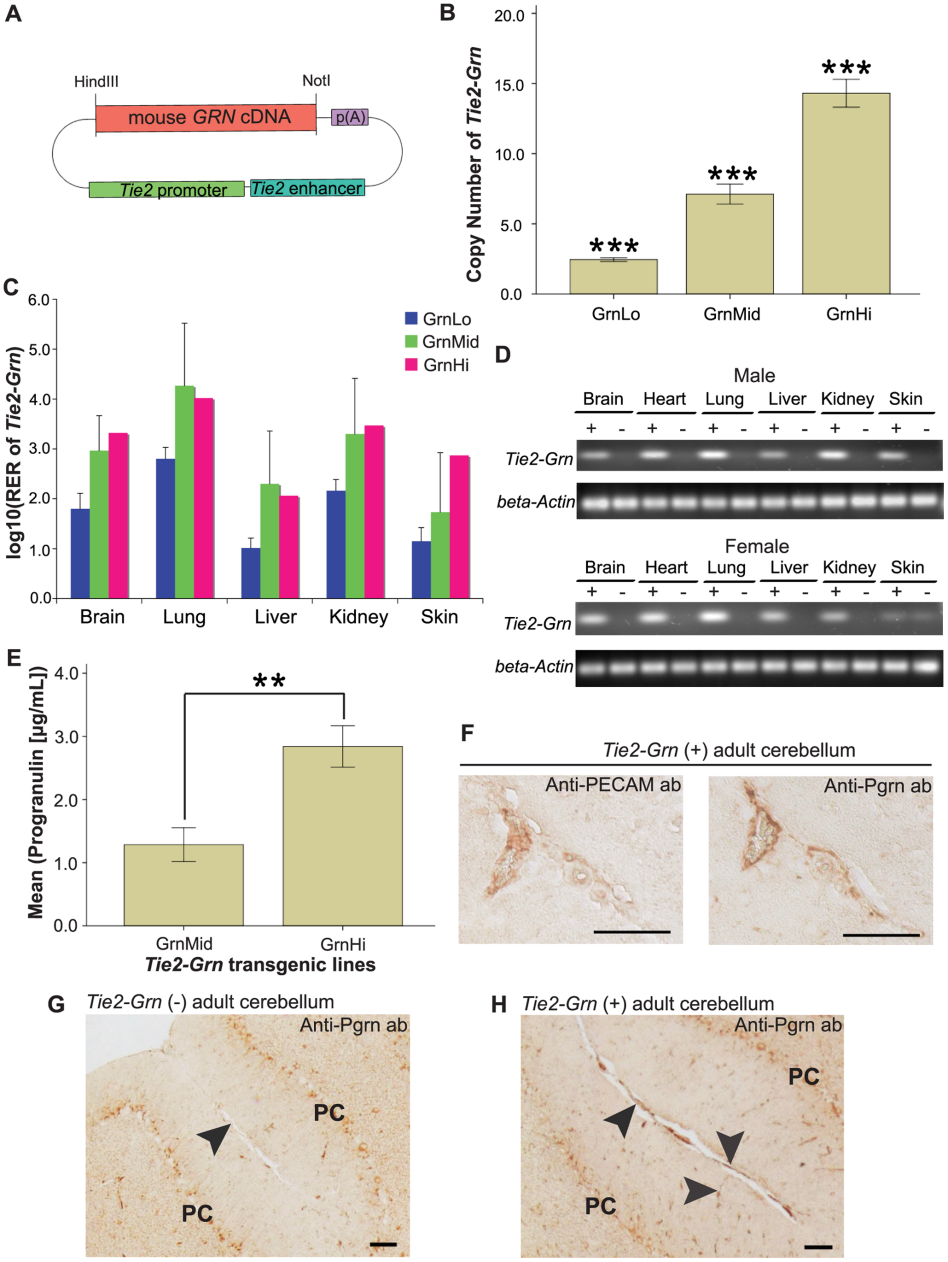
Characterization of the *Tie2-Grn* mouse model. (A) Schematic of the *Tie2-Grn* targeting vector. (B) PCR-based quantification of the *Tie2-Grn* copy number in the three *Tie2-Grn* lines. N = 3 for each line, error bars denote +/− S.E. One-Way ANOVA indicated significant difference between groups (3 lines) at the 0.001 level. (C) qRT-PCR of *Tie2-Grn* mRNA in adult (3 months old) mouse tissues expressed as log_10_ (Relative Expression Ratio) calculated as the expression in the *Tie2-Grn* positive mice divided by baseline non-specific amplification and normalized to *β-actin* from *Tie2-Grn* negative tissues. Error bars denote +/− S.E. N = 8 (GrnLo), N = 3 (GrnMid), N = 1 (GrnHi). (D) PCR products for the *Tie2-Grn* mRNA analyzed by agarose gel, demonstrating the specificity of the amplification reaction for the *Tie2-Grn* mRNA and lack of product in the *Tie2-Grn* negative tissues. (E) Progranulin protein serum levels in the GrnMid and GrnHi line. Error bars denote +/− S.E. N = 4 (GrnMid) and N = 5 (GrnHi). Independent samples T-test revealed significant difference, *p* = 0.008 (two-tailed). (F) PECAM, a marker for endothelial cells, and progranulin immunostaining overlaps in the adult cerebellum. Immunostaining for progranulin in the adult cerebellum from a *Tie2-Grn* negative mouse (G) and a positive littermate (H). Capillaries are indicated by arrowheads. Note also that some neurons, such as Purkinje cells (PC) stain for progranulin. The scale bar indicates 20 µm in all sections. Asterisks indicate statistical significance in 1B and E; significance of <0.001 is ***, <0.01 is ** and <0.05 is *.

The *Tie2-Grn* gene (Fig. 1A) was detected in 3 of 47 mice tested. We estimated that *Tie2-Grn* transgenic line 1 (female founder MIL-535) had 2–3 copies (2.45±0.23), *Tie2-Grn* transgenic line 2 (male founder MIL-772) had 13–16 copies (14.3±1.72), *Tie2-Grn* transgenic line 3 (female founder MIL-794) had 6–8 copies (7.12±1.23) respectively (Fig. 1B). Line 1 was subsequently referred to as GrnLo, line 3 as GrnMid, and line 2 as GrnHi to reflect their respective copy numbers. The three *Tie2-Grn* founders appeared healthy and showed no obvious gross external abnormalities.

### Expression of the *Tie2-Grn* Transgene in Transgenic Mice

The founder lines were backcrossed on a C57BL/6 background. The expression of *Tie2-Grn* mRNA was established in various mouse tissues by real time qPCR (Fig. 1C). The transgene was expressed in all the organs tested, with the highest levels detected in the lungs. Agarose gel analysis of the amplified PCR products confirmed that the *Tie2-Grn* was detected only in the transgenic mice (Fig. 1D). Progranulin protein levels were higher in the *GrnHi* positive animals compared to the *GrnMid* positive animals (Fig. 1E). Serial sections stained for progranulin and the endothelial marker protein PECAM (CD31) show overlay of progranulin with endothelial cells in *Tie2-Grn* positive mice (Fig. 1F). Over-production of progranulin by endothelial cells in the *Tie2-Grn* positive mice was demonstrated by immunohistological staining (Fig. 1G and H). This confirms that the *Tie2-Grn* transgene has a widespread tissue distribution, and results in a stable increase in progranulin production in endothelial cells.

### Higher Mortality in Transgenic Litters

**Figure 2:**
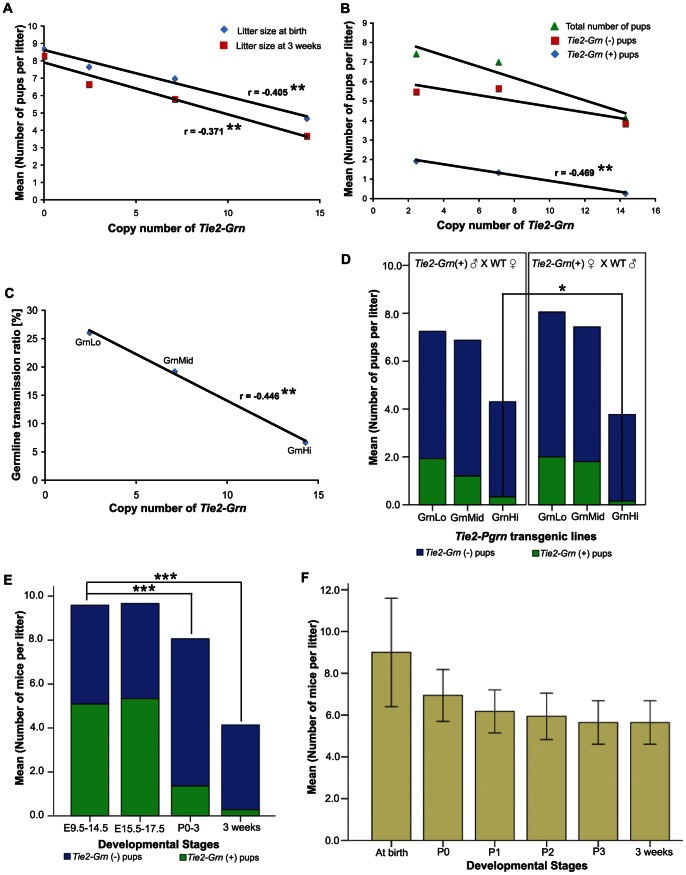
The *Tie2-Grn* transgene results in elevated mortality during perinatal period. (A) Mean litter size at birth and weaning decreases as a function of higher *Tie2-Grn* copy number. N = 1220 pups. Correlation between litter size at birth and copy number is significant at the 0.01 level (2 tailed), R = −0.405. Correlation between litter size at weaning and copy number is significant at the 0.01 level (2-tailed), R = −0.371. (B) Comparison of survival statistics for the total number of pups per litter, *Tie2-Grn* negative pups per litter and *Tie2-Grn* positive pups per litter at weaning. N = 1069. Correlation is significant between *Tie2-Grn* positive litter size and copy number at the 0.01 level (2-tailed), R = −0.469. (C) Comparison of germline transmission at weaning of the *Tie2-Grn* gene expressed as a function of copy number number. N = 1069. Correlation is significant between germline transmission ratio and copy number at the 0.01 level (2-tailed), R = −0.446. (D) Litter sizes from matings with *Tie2-Grn* positive father or mother. Independent samples T-test was performed for individual transgenic lines. *p* = 0.260 (GrnLo); *p* = 0.095 (GrnMid). A slight maternal effect was observed in the GrnHi line (*p* = 0.041). (E) Litter sizes (*Tie2-Grn* positive and negative) throughout development. N = 653 pups, GrnHi line. Independent samples T-test for variable (%Tg) was conducted between groups E9.5–14.5 vs. E15.5–17.5 (*p = *0.8); E9.5–14.5 vs. P0–3 (*p*<0.001); and E9.5–14.5 vs. 3 weeks (*p*<0.001). (F) Death continued after birth (newborn), and the first, second and third days of life. Thereafter, most mice survived to weaning. Error bars denote +/−95% C.I. N = 17, GrnHi line. Asterisks indicate statistical significance in 2A–D; significance of <0.001 is ***, <0.01 is ** and <0.05 is *.

Mortality was higher in crosses between Tg × WT litters than in WT × WT litters both at birth and at three weeks. All three transgenic lines had lower litter sizes than corresponding WT × WT crosses. Figure 2A shows that the average litter size drops from 8.7 at birth for wildtype crosses to 4.7 for the GrnHi crosses. The average litter sizes, both at birth and at three weeks, showed a statistically significant inverse linear correlation with respect to the copy number of *Tie2-Grn* in the three transgenic lines (Fig. 2A). A comparison of litters that were genotyped at three weeks of age (Fig. 2B) indicates that the diminishing litter sizes were due specifically to lower numbers of mice carrying the *Tie2-Grn* gene rather than a non-specific effect on survival regardless of genotype. The germline transmission ratios of *Tie2-Grn* positive mice at three weeks were calculated as 26.01%, 19.61% and 4.72% for GrnLo, GrnMid and GrnHi respectively (Fig. 2C), and show an inverse linear correlation with *Tie2-Grn* copy number. Assuming a predicted germline transmission of 50% based on a Tg × WT breeding scheme, this indicates losses of approximately half, three fifths and nine tenths of the *Tie2-Grn* positive mice in the GrnLo, GrnMid, and GrnHi lines respectively. This clearly shows a detrimental effect of *Tie2-Grn* on survival before or close to birth that is gene dosage-dependent. The survival of *Tie2-Grn* negative littermates was similar between GrnLo and GrnMid litters, while in the GrnHi litter, there was increased loss of *Tie2-Grn* negative mice. This suggested a possible maternal effect on the litter survival in the GrnHi line.

The maternal placenta is highly vascular, and defects in its development might influence the survival of litters. To investigate whether maternal effects influence survival of pups, we compared the survival ratios obtained from breeding either *Tie2-Grn* positive mothers or *Tie2-Grn* positive fathers with WT mice of the corresponding gender. Tests of correlation between the number of *Tie2-Grn* positive litter sizes (GrnLo, GrnMid and GrnHi lines) and two variables, gender of the transgenic parent and *Tie2-Grn* copy number, showed a statistically significant maternal effect for *Tie2-Grn* only in GrnHi litters. The maternal effect was not sufficient in magnitude to account for frequent loss of *Tie2-Grn* positive animals (Fig. 2D).

When we performed Tg × Tg crosses to obtain homozygous mice, we noticed several aborted litters from GrnHi × GrnHi crosses and GrnMid × GrnMid crosses. Among those litters that were not aborted, we observed multiple deformed newborns and at E17.5, the uterus showed identifiable resorption sites. Therefore, the homozygous crosses appeared to be present with earlier lethality. Given the difficulty in obtaining reliable live births in Tg × Tg crosses, all experiments were performed using Tg × WT crosses.

### Mortality occurs Predominantly in the Perinatal Period

Given that the GrnHi transgenic line shows the greatest premature mortality amongst the *Tie2-Grn* transgenic lines, we focused on this line to further evaluate the effect of the *Tie2-Grn* transgene on litter size throughout development. We collected and genotyped embryos pooled at between embryonic days E9.5 to E14.5, later fetuses pooled at between E15.5 and E17.5, neonates (postnatal days 0–3), and young mice at three weeks. As before, an under representation of *Tie2-Grn* positive mice at birth and at weaning was observed (Fig. 2E). In contrast, in the E9.5–14.5 embryos and E15.5–17.5 fetuses, the ratio of *Tie2-Grn* positive to negative animals is close to 1∶1. Death continues postnatally until day three (Fig. 2F). *Tie2-Grn* positive pups that survive beyond the third day after birth are likely to be alive at three weeks. Clearly, therefore, the lethality of the *Tie2-Grn* transgene occurs close to birth.

### Abnormal Coloration and Subcutaneous Hemorrhage in *Tie2-Grn* Positive Mice

**Figure 3:**
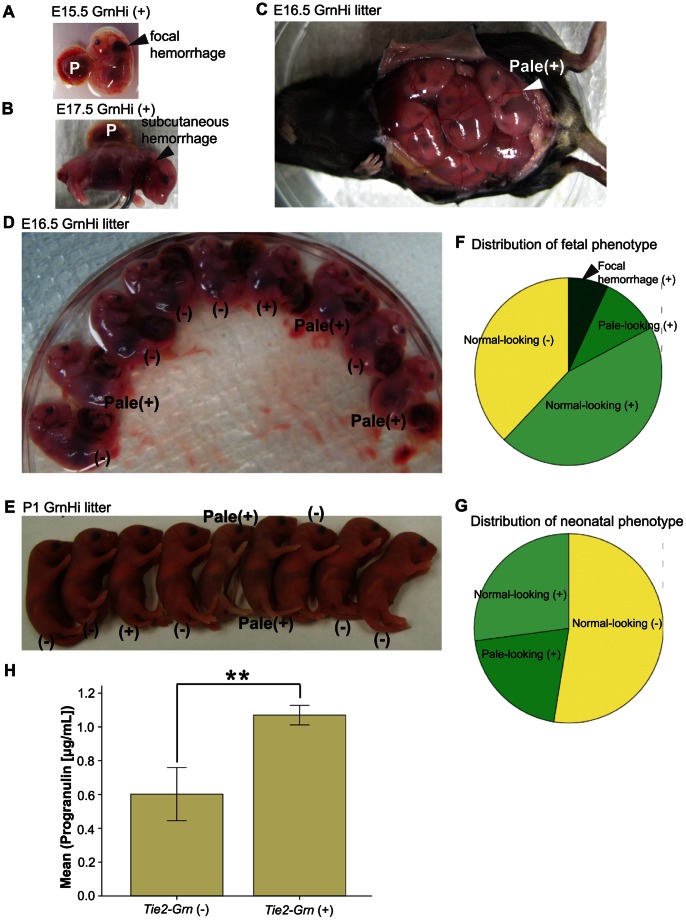
*Tie2-Grn* dependent abnormalities. (A–D) Appearance of embryos from E15.5 to E16.5 showed subcutaneous hemorrhage, often behind the head, (indicated by arrowheads, P is placenta) (A and B) or pale appearance (C and D) (indicated by arrowhead in C). Litters were genotyped either *ex utero* (D) or post-natally (E). Externally visible abnormalities were found only in *Tie2-Grn* positive mice. The phenotype is variable, as some *Tie2-Grn* positive mice were indistinguishable from the negative littermates. The genotype is indicated in the figure by (+) or (−). (F) The distribution of externally visible abnormalities in E15.5–17.5 fetuses, N = 29 from the GrnHi line and, (G) from P0–P1 live neonates, N = 59 from the GrnHi line. The neonates found dead were not included. (H) Levels of progranulin protein were higher in the amniotic fluid of the *Tie2-Grn* positive fetuses than negative fetuses (E16.5). Independent samples T-test showed significant difference between the transgenic and negative population, *p* = 0.007 (two-tailed). Errors bars denote +/− S.E. N = 6 from the GrnHi line only. Asterisks indicate statistical significance in 3H; significance of <0.01 is indicated by **.

There are a number of abnormalities in the external appearance of the *Tie2-Grn* positive fetuses and neonates in the GrnHi and GrnMid lines. Figure 3 shows a representation of these abnormal mice from the GrnHi line. These were first observed from E15.5 when a subset of *Tie2-Grn* positive mice showed subcutaneous focal hemorrhage or ruptured vasculature (Fig. 3A and B). At E16.5, some *Tie2-Grn* positive fetuses observed *in utero* had a pale appearance (Fig. 3C and D). Pale fetuses and pale neonates were collected from both the GrnMid (not shown) and the GrnHi line but were more frequently noted in the GrnHi line (Fig. 3D and E). Finally, a subset of *Tie2-Grn* positive embryos appeared indistinguishable from *Tie2-Grn negative* littermates with respect to external appearance (Fig. 3D and E).

Some pallid *Tie2-Grn* positive fetuses were carried to term and delivered alive (Video S1), thus pale coloration preceded death. Pallor was more immediately obvious at birth than *in utero.* Externally visible subcutaneous hemorrhages were not observed in mice at birth suggesting that these fetuses may have succumbed to hemorrhaging *in utero*. Visual observation revealed that certain live-born pallid mice had apparent respiratory difficulty and died shortly after birth (Video S2). The frequencies of externally evident abnormalities for embryos and neonates are summarized in figures 3F and 3G. The pale coloration might be due either to anemia or vascular malformation. We therefore evaluated red blood cell count, and although the red blood cell count was slightly lower in *Tie2-Grn* positive embryos, this did not correlate with pallor (Fig. S1). The overexpression of endothelial progranulin significantly increased secreted progranulin levels in the amniotic fluid of *Tie2-Grn* positive fetuses compared to the negative littermates at E15.5 (Fig. 3H).

Although *Tie2-Grn* mRNA is expressed in adult organs (Fig. 1C and D) some transgenic mice survived to maturity. No gross morphological differences were seen among survivor *Tie2-Grn* positive mice when compared with WT mice, at birth or at weaning. Overall, *Tie2-Grn* positive survivor mice had equivalent body weights at 3 weeks as their *Tie2-Grn* negative counterparts except in GrnHi litters, where the WT littermates were heavier (Fig. S2). The difference is relatively small but statistically significant. Subsequent analysis, showed, however, that the major determinant of weight at 3 weeks is litter size rather than genotype, with WT littermates from GrnHi litters gaining weight because of small litter size (Table S1). We investigated the structure of blood vessels and progranulin expression in the adult survivor mice (Fig. S3). Blood vessels in positive and wildtype littermates appeared indistinguishable with respect to diameter and coverage with a well-developed vascular smooth muscle cell layer (Fig. S3A–D). In the *Tie2-Grn* positive adult survivor mice, endothelial cells stained for progranulin (Fig. S3C) but less robustly than observed in the*Tie2-Grn* positive fetuses, and fewer progranulin positive vessels (Fig. S3E) were observed in the positive adults than in the fetuses and neonates. This may contribute to the lack of vascular abnormalities in the transgenic adults.

### Vascular Abnormalities in the Neonatal *Tie2-Grn* Positive Mice

**Figure 4:**
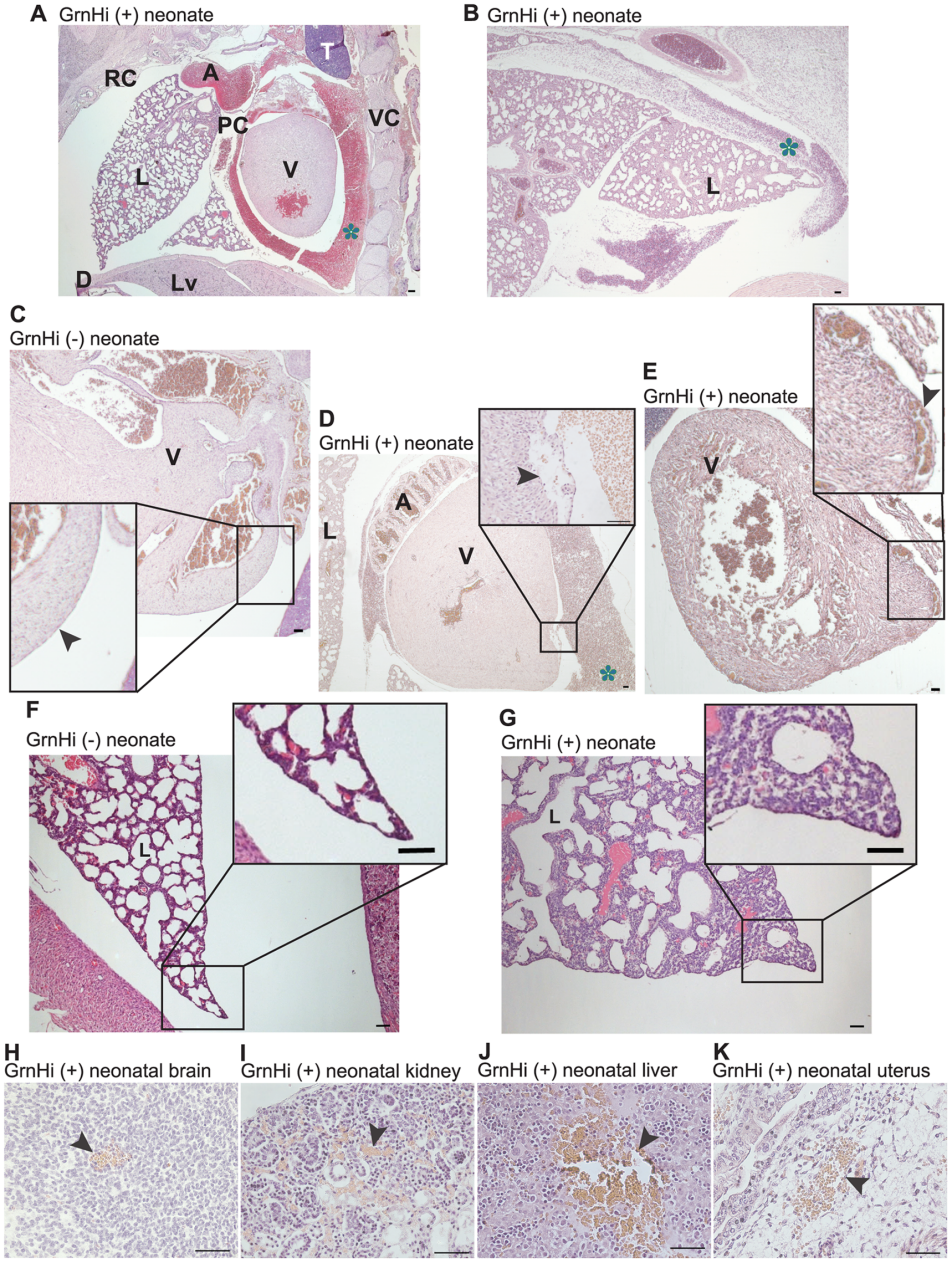
Vascular abnormalities and altered lung structure in *Tie2-Grn* positive neonatal mice. Extravasated blood (*) in the pericardial cavity (A) and in the pleural cavity adjacent to the lung (B) of *Tie2-Grn* positive neonates. (C) The heart from a *Tie2-Grn* negative neonate; note the absence of blood in the pericardial space. The insert shows a higher power view of the normal epicardial wall (arrow). (D) Blood pooling in the pericardial cavity of a*Tie2-Grn* positive neonate; note the ruptured vessel shown at higher power magnification in the insert (arrow). The neonates shown in (A–D) were collected alive, the transgenic neonates in A, B and D died soon after collection. (E) The heart from a post-mortem *Tie2-Grn* positive neonate; note the absence of bleeding into pericardial space but the presence of enlarged epicardial vessels (arrow) in the ventricle wall, shown at higher power magnification in the insert. Appearance of the lungs in a (F) *Tie2-Grn* negative neonate and (G) a matched *Tie2-Grn* positive neonate. Note that the alveolation is incomplete in the *Tie2-Grn* positive neonate with small alveoli and thickened mesenchymal alveolar wall (G). Localized hemorrhages (arrows) were observed in multiple organs of *Tie2-Grn* positive mice at birth including (H) brain, (I) renal cortex, (J) liver, and (K) uterus. All sections stained with H&E. Scale bars denote 20 µm. Animals were obtained from the GrnHi line. Abbreviations used: A, atrium; D, diaphragm; L, lung; Lv, liver; PC, pericardial cavity; RC, rib cage; T, thymus; V, ventricle; VC, vertebral column.

We investigated the histological basis of perinatal mortality in *Tie2-Grn* positive mice and observed a number of vascular abnormalities of varying severity. Massive internal bleeding was detected in a number of the *Tie2-Grn* positive mice (Fig. 4A, B and D) but not in *Tie2-Grn* negative mice (Fig. 4C). Figures 4A and 4D show *Tie2-Grn* positive neonates that were collected alive but died shortly after birth. Both succumbed to cardiac tamponade, where blood collected around the heart in the pericardial cavity. An abnormal epicardial vessel with the appearance of rupture that may have contributed to the blood hemorrhage was observed in the ventricle wall from a *Tie2-Grn* positive neonate with cardiac tamponade (Fig. 4D). Epicardial vessels were enlarged in some mice (Fig. 4E) compared to the *Tie2-Grn* negative counterparts (Fig. 4C) even when cardiac tamponade did not precede post-natal death. Blood hemorrhage was also observed in the pleural cavities of some GrnHi positive neonates (Fig. 4B). Intriguingly, the interstitial wall of the *Tie2-Grn* positive neonatal lung appears to be hypercellular (Fig. 4G) compared to the *Tie2-Grn* negative counterpart (Fig. 4F).

Bleeding into body cavities was not detected in all *Tie2-Grn* positive neonates; however, widely disseminated local internal hemorrhage affected several organs, including the brain, the kidney, the liver and the uterus (Fig. 4H–K). The affected organs were not always the same in every *Tie2-Grn* positive neonate. Some of the *Tie2-Grn* positive neonates did not have any observable abnormalities; however, since it is not possible to sample all regions exhaustively, it is likely that small, localized hemorrhages may have been missed in some apparently normal *Tie2-Grn* positive neonatal mice. Other than vascular abnormalities, the tissues examined appeared to be normal. Regardless of the organs affected, the hemorrhages and the enlarged vessels demonstrate that the blood vessels in *Tie2-Grn* positive fetuses and neonates were abnormal and prone to rupture.

### Structurally Intact and Functional Vasculature in Early *Tie2-Grn* Positive Embryos

**Figure 5:**
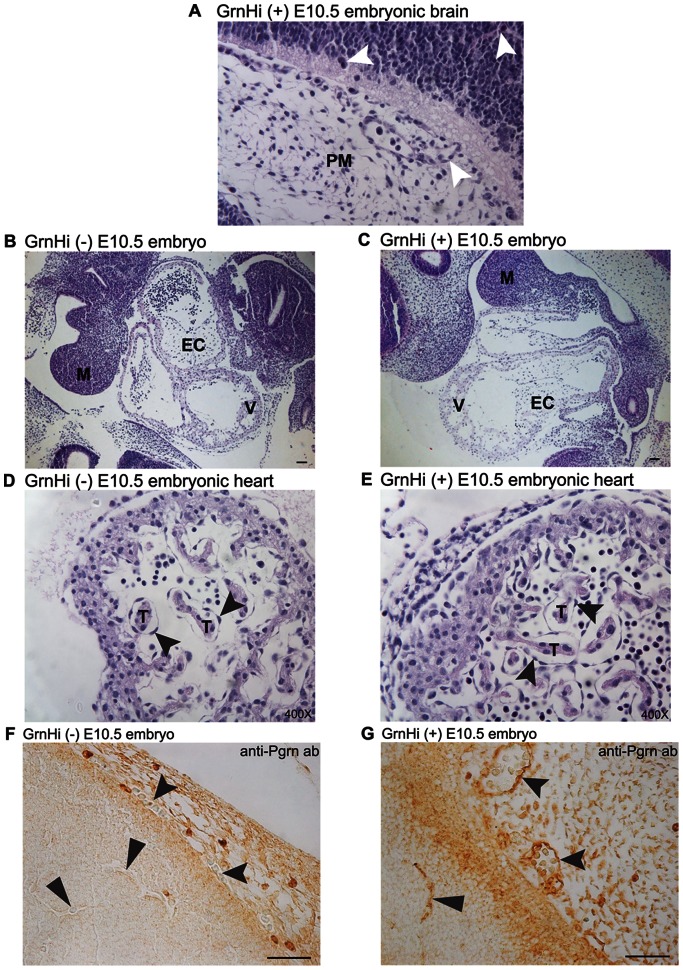
The development of the vasculature and heart appears normal at mid- gestational *Tie2-Grn* positive embryos (E10.5). (A) Sprouting capillaries (white arrow heads) in the brain and meninges of a *Tie2-Grn* positive embryo (PM, pia mater). (B) and (C) illustrate the appearance of the heart in a *Tie2-Grn* negative (B) and *Tie2-Grn* positive embryo (C), which are structurally similar. EC, endothelial cushion. (D–E) Trabeculation and the formation of the endothelial lining of the trabeculae are indistinguishable in a *Tie2-Grn* negative (D) and *Tie2-Grn* positive embryo (E) embryos. (Black arrow heads point to the endothelium covering the cardiac muscle of the trabeculae). A *Tie2-Grn* negative (F) and *Tie2-Grn* positive embryo (G) stained for progranulin demonstrate the increased production of progranulin in the endothelia (black arrow heads) of the *Tie2-Grn* positive embryo at E10.5. M, mandible; EC, endocardial cushion; T, trabeculae. Scale bars denote 20 µm. Images were obtained from the GrnHi line.

**Figure 6:**
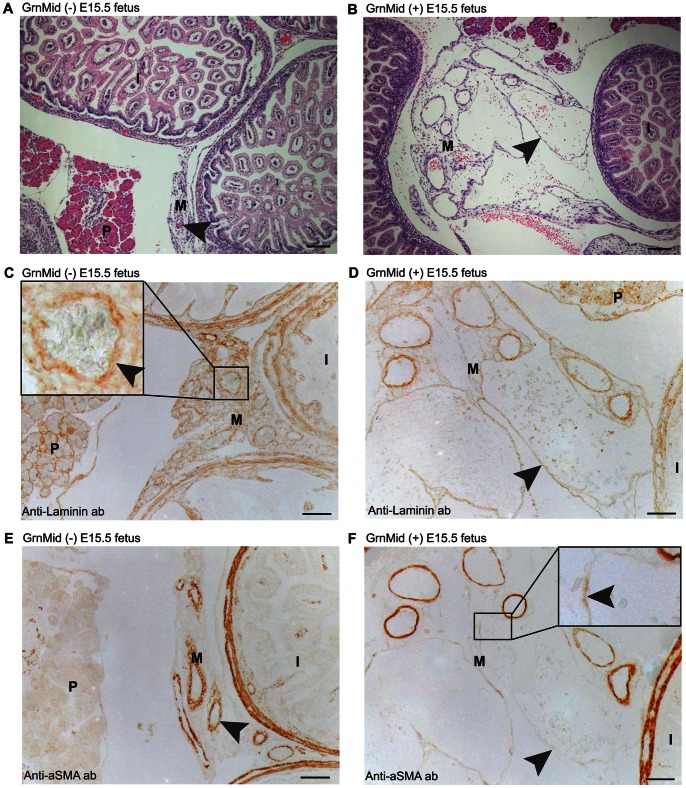
Vascular abnormalities were first observed at E15.5 in the *Tie2-Grn* positive embryos. A comparison of mesenteric vessels (black arrow heads) in (A) a *Tie2-Grn* negative and (B) a *Tie2-Grn* positive fetus stained with H&E. Note the presence of highly distended vessels in (B). Blood vessels stained strongly for laminin, a basement membrane protein in *Tie2-Grn* negative fetuses (insert, black arrow head) (C), but in *Tie2-Grn* positive fetuses (D) laminin stained weakly, especially in the most distended vessels. Mesenteric vessels from *Tie2-Grn* negative fetuses (E) showed extensive coverage with smooth muscle α-actin positive mural cells, while in *Tie2-Grn* positive fetuses (F) distended vessels were in large part negative for smooth muscle α-actin positive mural cells (black arrow heads). P, pancreas; I, intestines; M, mesentery. Scale bars denote 20 µm. Images were obtained from the GrnMid line.

Given that *Tie2-Grn* positive mice show elevated mortality around birth, we sought to establish the time of onset of *Tie2-Grn* abnormalities during development. To trace back the initial time-point of the disruption in blood vessel formation of the *Tie2-Grn* positive mice, we investigated mice from the GrnHi line in mid gestation from E10.5–14.5 (Fig. 5) and late gestation from E15.5–17.5 (Fig. 6). *Tie2-Grn* positive E10.5 embryos exhibited normal endothelial sprouting (Fig. 5A). Cardiac development appeared normal. Endocardial cushions were properly formed (Fig. 5B and C). Since endocardial cushion are formed when endothelial cells undergo an endothelial to mesenchymal transition, the transdifferentiation of the endothelial cells during development must occur normally in the *Tie2-Grn* positive embryos. Endothelial lining of the trabeculae of the heart in the *Tie2-Grn* positive embryos appeared similar to that of the *Tie2-Grn* negative counterpart (Fig. 5D and E). Figures 5F and 5G confirmed the over-production of progranulin at E10.5. Immunohistochemical analysis of the embryonic aortic vessel revealed strong expression of progranulin in the endothelial lining, strong expression of laminin in the basement membrane underlying the endothelial cells and a thick band of vascular smooth muscle cells enveloping the aorta as revealed by robust smooth muscle α-actin staining (not shown). We conclude therefore that the *Tie2-Grn* transgene has no overt effect on early vasculogenesis and angiogenesis, nor does it interfere with embryonic endothelial functions in endocardial cushion formation and consequently valve formation.

### Enlarged Blood Vessels with Abnormally Sparse Mural Cell Coverage in Late *Tie2-Grn* Positive Fetuses

**Figure 7:**
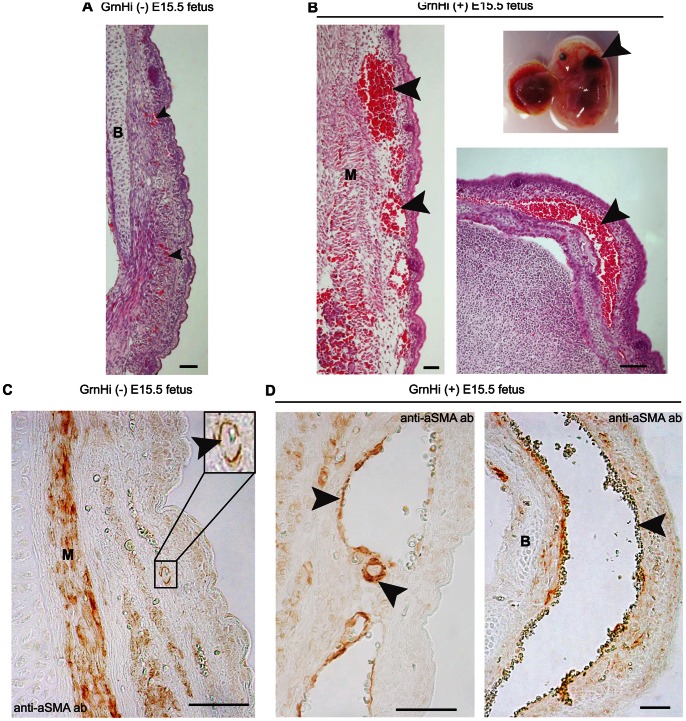
Compromised subcutaneous vessel structure at E15.5 in the *Tie2-Grn* positive embryos. Some *Tie2-Grn* positive fetuses exhibited subcutaneous hemorrhage at E15.5, particularly in the head region. (A) The scalp from a *Tie2-Grn* negative littermate and (B) from a *Tie2-Grn* positive littermate showing highly enlarged vessels not present in the negative counterpart. Sections were stained with H&E, the arrow heads point to blood vessels; the insert shows the external appearance of the *Tie2-Grn* positive fetus with the arrow head showing the extent of subcutaneous hemorrhage. The small vessels of the *Tie2-Grn* negative fetus (C) are enclosed in smooth muscle α-actin positive mural cells (insert). (D) The smaller vessels in the *Tie2-Grn* positive fetus is enclosed smooth muscle α-actin positive mural cells but the more enlarged vessels show incomplete association with smooth muscle α-actin positive mural cells (arrow heads indicate vessels). B, bone; M, muscle. Scale bars denote 20 µm. Images were obtained from the GrnHi line.

Compromised vasculature was first observed in *Tie2-Grn* positive fetuses at E15.5, both in the GrnMid and GrnHi lines (GrnLo mice were not investigated). As reported in figure 3, visual inspection of *Tie2-Grn* positive fetuses revealed some that were pale looking and some that harbored subcutaneous hemorrhages (Fig. 3A–D). Upon histological analysis, enlarged blood vessels were observed in the mesentery, the heart, the lung and the head (the meninges and the scalp) of *Tie2-Grn* positive fetuses from the GrnMid and GrnHi transgenic lines. Examples are shown in figures 6 and 7 from the mesentery and scalp respectively. The mesenteric vessels of *Tie2-Grn* positive embryos are unusually large compared to *Tie2-Grn* negative littermates (Fig. 6A and B). Mesenteric vessels of *Tie2-Grn* negative embryos display a well-developed basement membrane, as indicated by the strong staining for laminin (Fig. 6C) and well-defined mural cell investiture, as defined by the presence of smooth muscle α-actin positive cells (Fig. 6E). In comparison, in the *Tie2-Grn* positive embryo, although smaller mesenteric vessels retain laminin and smooth muscle α-actin staining, the highly enlarged vessels have a weakened layer of basement membrane (Fig. 6D) and an abnormally sparse layer of vascular smooth muscle mural cells (Fig. 6F).

Given that visual inspection of fetuses at E15.5 showed that some had subcutaneous hemorrhaging, especially around the head region, we examined the subcutaneous vessels in *a Tie2-Grn* positive E15.5 fetus that had a subcutaneous hemorrhage near the back of the neck region (Fig. 7). Normal subcutaneous vessel structure from a *Tie2-Grn* negative E15.5 embryo is shown in figure 7A. The *Tie2-Grn* positive embryo showed dilated and ruptured scalp vessels (Fig. 7B). Immunohistochemical studies (Fig. 7C and D) revealed that while the vascular smooth muscle layer coating the blood vessels is present in certain small blood vessels it is absent or depleted from large regions of the disrupted blood vessel in the *Tie2-Grn* positive embryo (Fig. 7D). In general, blood vessel enlargement was more prominent in the *Tie2-Grn* positive fetuses with externally visible subcutaneous hemorrhages than in the pale-looking *Tie2-Grn* positive fetuses.

**Figure 8:**
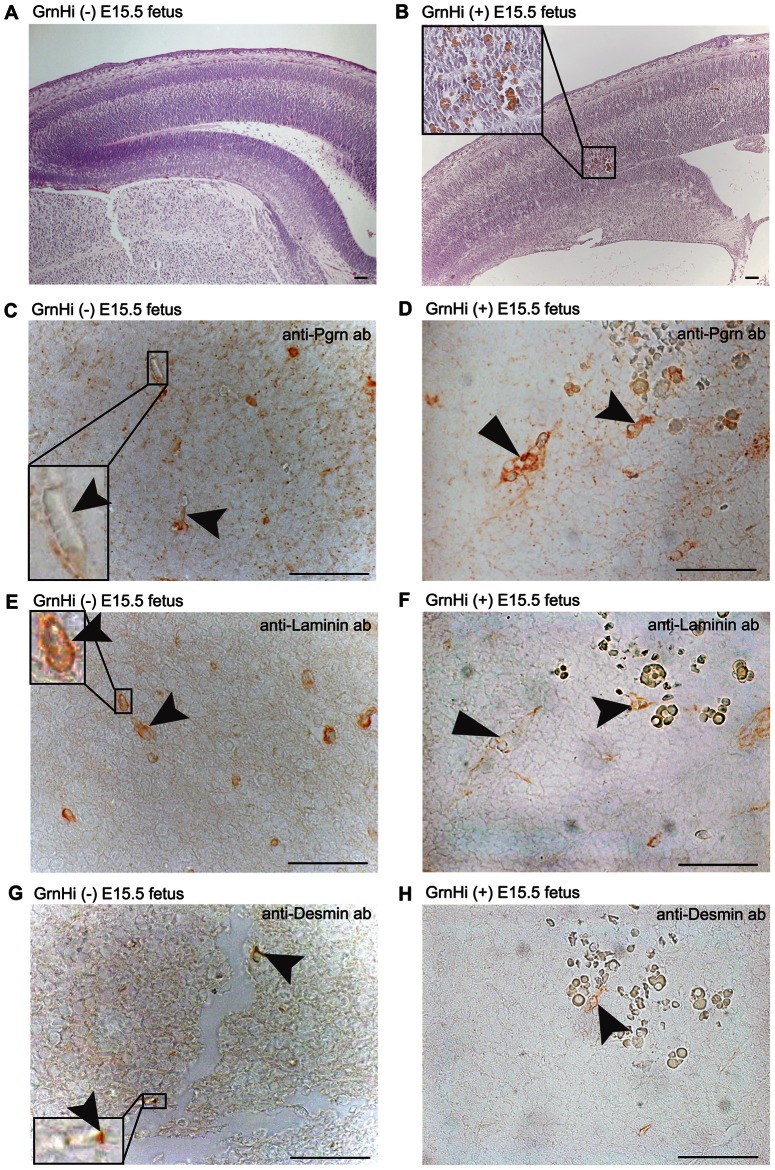
Cerbrocortical hemorrhaging in *Tie2-Grn* positive fetus at E15.5 associated with reduced basement membrane and pericyte enclosure of capillaries. H&E staining of the cerebral cortex of (A) a *Tie2-Grn* negative fetus with no hemorrhage sites and (B) a *Tie2-Grn* positive fetus. Small localized hemorrhages were observed in the cerebral cortex of the *Tie2-Grn* positive fetus (insert) but not the *Tie2-Grn* negative fetus. Capillaries (black arrow heads) were poorly stained for progranulin in the *Tie2-Grn* negative fetus (C), but displayed high levels of progranulin in the *Tie2-Grn* positive fetus (D). The basement membrane protein laminin clearly demarcated capillaries in the *Tie2-Grn* negative fetus (E, and insert). Some capillaries in the *Tie2-Grn* positive fetus showed strong laminin staining (tailed arrowhead) while other showed reduced laminin (flat arrowhead). Desmin, a marker for pericytes, was associated with capillaries in the *Tie2-Grn* negative fetus (F, and insert). Few capillaries in the *Tie2-Grn* positive were positive for desmin (arrowhead).

Focal cerebral hemorrhages were observed at E15.5 in *Tie2-Grn* positive embryos (Fig. 8A and B). The endothelial lining of the blood vessels in the region of the hemorrhage of the *Tie2-Grn* positive fetus (Fig. 8D), but not *Tie2-Grn* negative fetuses (Fig. 8C) were strongly positive for progranulin. Capillaries in the *Tie2-Grn* negative control were uniformly lined with laminin (Fig. 8E). In the *Tie2-Grn* positive brain, some capillaries were also lined with laminin but others had an abnormally fine basement membrane, as marked by laminin staining, compared to the *Tie2-Grn* negative embryos (Fig. 8F), and were completely void of desmin-staining pericyte mural cells (Fig. 8G and H). Attenuation of the basement membrane and lack of mural cells is likely to have contributed to the onset of hemorrhaging.

### *Tie2-Grn* Positive Fetal Placenta Shows Subtle Abnormalities

While most quiescent adult endothelial cells do not display strong progranulin levels, the high expression of progranulin occurs as a normal physiological condition in some circumstances, in particular in the placenta (Fig. S4A and B). We examined the maternal and fetal components of the placenta at E15.5 from the GrnHi line for abnormalities. No vascular changes in the morphology of the placenta were detected, but we observed an increased number and much larger size of syncytial knots in *Tie2-Grn* positive fetal placentas, compared to the WT counterparts (Fig. S4C and D).

### PGRN and VEGF-A or VEGFR2 Expression

**Figure 9:**
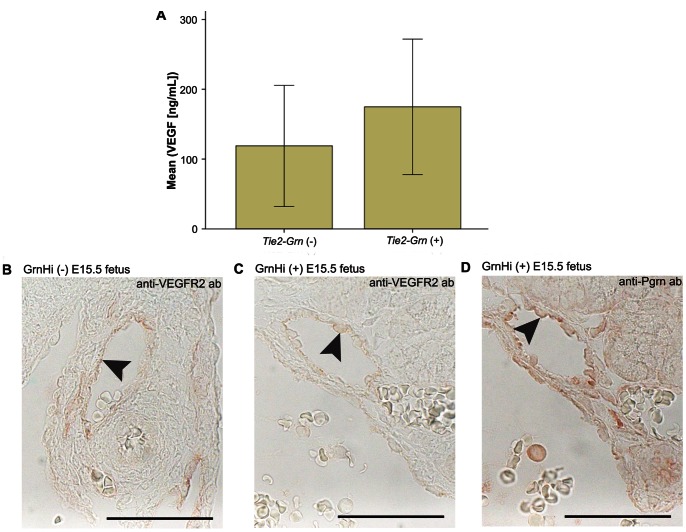
Relationship between *Tie2-Grn* expression and VEGF-A/VEGFR2 expression. (A) VEGF-A protein levels in the amniotic fluid. Independent samples T-test revealed no significant difference in VEGF-A levels between the *Tie2-Grn* positive fetuses and the *Tie2-Grn* negative fetuses (*p* = 0.423). Age of fetuses are E15.5–16.5. N = 8, GrnHi line. Error bars denote +/− S.E. VEGFR2 endothelial expression in (B) *Tie2-Grn* negative fetus and (C) *Tie2-Grn* positive fetus. Arrows show endothelial cells within the mesenteric vessels. (D) Serial sections of the *Tie2-Grn* vessel in (B) expressed progranulin. Scale bars denote 20 µm. Images were obtained from the GrnHi line.

In cancers, progranulin has been shown to increase VEGF levels which would stimulate angiogenesis. We were unable to detect immunoreactive VEGF-A expression associated with PGRN in tissue sections (not shown), and there was no significant increase in VEGF-A protein levels in the amniotic fluid of *Tie2-Grn* positive mice compared with non-transgenic littermates (Fig. 9A). VEGFR2, the major proangiogenic receptor for VEGF-A, was expressed at equivalent levels in vessels from *Tie2-Grn* positive mice and negative littermates (Fig. 9B–D).

## Discussion

There are many indications that progranulin might contribute to angiogenic processes [11] [24] [25] [26] [27] [28] [29]. It was however uncertain whether elevated progranulin levels in endothelial cells would influence vessel development and structure *in vivo*.

We addressed the question of the role that progranulin has in endothelial cells *in vivo* by developing mice with targeted endothelial expression of murine progranulin. The *Tie2* gene is expressed throughout vascular development, including in angioblasts, and in sprouting blood vessels [33]. A *Tie2*-promoter-enhancer construct supports the expression of heterologous genes in endothelial cells during embryonic development and in adults [34] [35]. Three *Tie2-Grn* mouse lines were developed, with different *Tie2-Grn* copy numbers. The *Tie2-Grn* transgene was reproducibly expressed in multiple tissues of transgenic adults with highest levels in the lung, which is among the most vascular of the tissues tested.

The expression of the *Tie2-Grn* gene was associated with an elevated risk of perinatal death in all three *Tie2-Grn* mouse lines. Mortality and transmission of the *Tie2-Grn* gene correlated inversely with *Tie2-Grn* copy number, with greatest mortality and lowest germ line transmission observed in the GrnHi litters. Thus, *Tie2-Grn* mortality is reproducible between different transgenic lines and is gene dosage-dependent. The death of the *Tie2-Grn* positive animals occurred at or around birth, then continued for three days after birth, after which time those *Tie2-Grn* positive neonates that remained alive would generally survive to maturity and appeared fully viable. Deaths were caused by vascular abnormalities of varying severity that included massive internal bleeding into body cavities, localized hemorrhage within organs, enlarged vessels with thin walls, and apparent vessel rupture in tissues, including the epicardium (Fig. 4D). Taken together, elevated endothelial expression of progranulin has a profound effect on vascular formation and integrity.

The *Tie2-Grn* positive mice that survived continued to transmit the phenotype of vascular defects to their offspring but displayed no obvious vascular abnormalities. Adult *Tie2-Grn* expression appears less active than in the fetuses and neonates (Fig. S3). Phenotypic variability and incomplete penetration with elevated transgene expression, as occurred with *Tie2-Grn*, is observed in mice even with a pure genetic background [36], and the underlying causes have been discussed [37].

Vasculogenesis, that is, the *de novo* formation of vessels and their subsequent maturation to form a capillary nexus with mural cell recruitment occurs between E6.5 and E9.5 in mice [38]. The earliest embryos examined, E10.5, showed normal vasculature and evidence for normal capillary sprouting and endothelial lining of cardiac trabeculae. Cardiac development appeared normal. Progranulin was overexpressed in endothelial cells of *Tie2-Grn* positive embryos at this time. During development some endothelial cells undergo a transition to mesenchymal cells, for example in the formation of endocardial cushions, structures which will later develop into heart valves [39] [40]. The endocardial cushions of *Tie2-Grn* positive pups are fully formed, indicating normal endothelial-mesenchymal transition. Clearly, therefore, the absence of any vascular phenotype at E10.5 in *Tie2-Grn* positive embryos precludes a major role for progranulin during vasculogenesis. At present, it is unclear why progranulin has little effect on early vascular development, given the dramatic actions of progranulin later in gestation. One possibility is that receptors or cofactors that modulate progranulin action are missing at the earlier stages. Definitive receptors for progranulin have not been identified (review in [14]) although progranulin binds other receptors such as the TNFα receptor [21] and the Toll-like receptor 9 [22]. Progranulin is, however, known to interact with domain V of perlecan, (basement membrane-specific heparan sulfate proteoglycan) [41], a protein whose expression in endothelial cells commences around E10.5 [42]. Domain V of perlecan promotes endothelial cell angiogenesis but inhibits vascular smooth muscle cell growth [43] and its interaction with progranulin has been suggested to regulate tumor angiogenesis [26].

The earliest vascular abnormalities occurred at E15.5. Focal hemorrhages colocalized with reduced basement membrane deposition and limited mural cell recruitment and, as in the neonatal vessels, the fetal vessels were enlarged and disorganized. Some vessels, however, had high levels of progranulin, but were, nevertheless invested with a well-formed basement membrane and mural cell envelope. These tended to be smaller than the vessels that lacked well defined mural cell coverage. Their presence demonstrates that the production of progranulin by endothelial cells is not, by it itself sufficient to prevent mural cell recruitment. Progranulin activates endothelial proliferation and migration *in vitro* [11] suggesting that the vessel dilation seen in *Tie2-Grn* mice is, at least in part, due to a direct action of progranulin on the endothelia. Importantly, however, mural cells exert a suppressive effect on endothelial angiogenesis, and their absence results in endothelial hyperplasia and microaneurysms [44] [45]. It is likely, therefore, that the lack of mural cell restraint in thin walled *Tie2-Grn* positive vessels would amplify progranulin-mediated endothelial vessel enlargement, and contribute significantly to the disruption of vessel integrity seen in *Tie2-Grn* positive mice from E15.5 onwards. The production of progranulin in endothelial cells is tightly regulated in wildtype mice [11] [23] [24], which presumably limits progranulin angiogenic action under physiological conditions only to situations where it is required.

Other than vascular defects, the tissues appear mostly normal with a few exceptions. Some *Tie2-Grn* positive neonates had hypercellular alveolar walls indicative of underdevelopment of the alveolation process. This may be due to the increased circulating progranulin. Pulmonary angiogenesis is critical in alveolation and its disruption in late development results in a marked inhibition of alveolation [46]. The placenta, in which the maternal vessels have high levels of progranulin even in wild-type mice showed no structural alterations in the *Tie2-Grn* positive mice, but did exhibit increased numbers and size of syncytial knots (Fig. S4). The syncytial knots are indicators of placental maturity, and their number increases following placenta hypoxia [47] [48] [49]. The non-vascular features of the *Tie2-Grn* positive phenotype are, therefore, likely to be secondary indications of abnormal blood vessel development or function.

**Figure 10:**
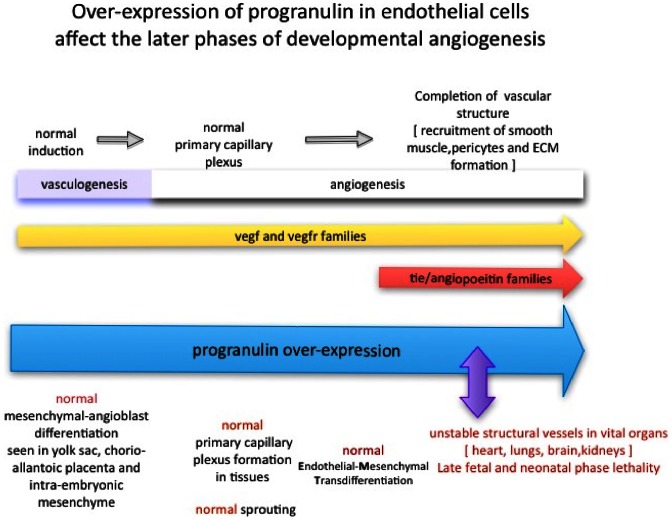
Summary of the effect of expression of progranulin during vascular development. The upper bar summarizes major landmarks in vascular development. VEGF and its receptors (yellow bar) are required for early vasculogenesis and for angiogenesis throughout development. Tie2 and its ligand angiopoietin (red bar) are required for cardiac development and for vessel stabilization, but not for early vasculogenesis (see text for details). When progranulin was expressed developmentally (purple bar) it had no effect on vasculogenesis, the formation of the primary capillary plexus, sprouting or endothelial-mesenchymal transition. Its major actions were on vessel size and structural stability in late embryonic and neonatal phases. The timing of its actions is therefore distinct from those of the well-established angiogenic growth factors, suggesting that its effect on vessel growth and stability constitutes a novel angiogenic pathway.

Figure 10 summarizes the timing of *Tie2-Grn* action on the blood vasculature, comparing it to the landmarks in vascular development and the action of critical regulatory proteins in these processes. Genetic disruption of VEGFR2 [50] or VEGF [51] [52] results in a failure of vasculogenesis. Deletion of angiopoietin-1, the ligand for Tie2, does not impede vasculogenesis but results in cardiac abnormalities with failure to form trabeculae by E9.5, and poor stabilization of blood vessels [53]. Loss of PDGF-beta or its receptor, PDGF-receptor β, cause a failure in mural cell recruitment in mid-gestation [45]. Progranulin acted only late in fetal life, suggesting that it regulates unique aspects of angiogenesis distinct from the well-established angiogenic factors.

Progranulin is expressed in tumor vasculature [25] [26] [27] [28] [29]. Strong vascular progranulin expression correlated with tumor recurrence in astrocytoma and with decreased patient survival in glioblastomas [29]. Cancer cells also overproduce progranulin, and this has been associated with increased VEGF production [27] [28] [30] [31]. In the *Tie2-Grn* positive mice, however, there were no changes in VEGF levels or changes in VEGFR2 expression. Progranulin stimulates endothelial cell proliferation and migration in culture [11], suggesting that it exerts angiogenic actions independently of VEGF. In a tumor, both mechanisms may occur, with progranulin produced by the tumor vasculature acting as an autocrine angiogenic factor, while progranulin produced by cancer cells may increase their VEGF secretion. The structure of tumor capillaries is often atypical, with large disorganized vessels that are poorly covered by mural cells [54]. This is strikingly similar to the abnormal vessels of the *Tie2-Grn* positive mice.

Taken together, there is strong support for a role for progranulin in angiogenesis and vessel formation. This may prove relevant in tumor angiogenesis, tissue repair and normal physiological tissue remodeling.

## Methods and Materials

### Ethics Statement

For experiments requiring animals, the mice were housed in facilities managed by the McGill University Animal Resources Centre. Animal experiments were conducted under a McGill University approved Animal Use Protocol (#5124) in accordance with guidelines established by the Canadian Council on Animal Care. Every effort was made to minimize suffering of the animals.

### Production of *Tie2-Grn* Targeting Vector

Mouse progranulin full-length cDNA plasmid, a RIKEN clone (clone# 2900053G23) pFLCI (modified Bluescript II) plasmid, was a gift from Max-Planck Institute for Biophysical Chemistry, Germany. A vascular endothelial promoter/enhancer package vector pSPTg.T2FpAXK (#52) was employed to make the *Tie2-Grn* transgene construct (gift from Montreal Clinical Research Institute (IRCM), originated from Dr. Sato, Texas Southwestern University at Dallas) [37]. This vector contains a *Tie2* promoter, a *SV40* polyA signal and a *Tie2* minimum enhancer fragment. The mouse progranulin cDNA was inserted in the multiple cloning site (MCS) located between the *Tie2* promoter and the polyA signal (Fig. 1A).

Since the *Tie2* vector contains the unique *HindIII* and *NotI* cloning sites, which are not present in the full-length *Grn* cDNA, *HindIII* and *NotI* were created at the ends of the *Grn* cDNA by PCR. Briefly, the mouse *Grn* cDNA was amplified by PCR (RIKEN clone as a template) using a forward primer containing a *HindIII* site and the *Grn* start codon, and a reverse primer containing a *NotI* site and the *Grn* stop codon. The *HindIII/NotI Grn* fragment was inserted into the pSPTg.T2FpAXK vector downstream of the *Tie2* promoter (Fig. 1A). The fidelity and accuracy of the PCR amplification and cloning was confirmed by DNA sequencing.

### Generation of *Tie2-Grn* Transgenic Mice

The new 5757 bp *Tie2-Grn* vector construct fragment was excised by *SalI* digestion and purified using a gel extraction kit (ZymoClean), followed by an Elutip column (Schleicher & Schuell), and the purified *Tie2-Grn* DNA fragment was used for microinjection into fertilized eggs from B6C3F1 mice (Jackson Laboratories) at the Transgenic Mouse Facility, IRCM. Three *Tie2-Grn* transgenic founder mice were picked out from screening 47 mice produced including two females (MIL-535, MIL-794) and one male (MIL-772).

The transgenic founder mice were identified by PCR of tail genomic DNA using transgene-specific primers and confirmed by DNA sequencing analysis.

Forward primer: AGCGGGAAGTCGCAAAGTTGTGAGTTGT;

Reverse primer 1: CTTGCCAAGAAGGTTGAGAAGCCATAC;

Reverse primer 2: AGAAATGCCCTTCTCCACACTCCA.

Both the reverse primers are paired up with the same forward primer. The forward primer is located on the *Tie2* promoter region of the transgene construct, whereas the reverse primers are located within the full-length mouse *Grn* cDNA region.

The housekeeping gene employed is mouse *β-actin* gene.

Forward primer: TGTGATGGTGGGAATGGGTCAGAA;

Reverse primer: TGTGGTGCCAGATCTTCTCCATGT.

Congenic strains were created by backcrossing the *Tie2-Grn* positive donors with the pure background inbred strain recipient mice (C57BL/6). The descendents of 5–10 generations after backcross can be used for phenotypic analysis [55]. Transgenic lines were established and maintained using this approach.

### *Tie2-Grn* Copy Number Estimation by Real Time qPCR

A quantitative real time PCR strategy was used to estimate the number of copies of *Tie2-Grn* transgene in each of the three transgenic lines, To selectively detect the *Tie2-Grn* transgene, we used primers that span exon junctions, since the *Tie2-Grn* transgene is intronless whereas the endogenous *GRN* has introns.

Forward primers: TCTTCTGGACACATGGCCTA (spanning the *GRN* exon 2–3 junction);

Reverse primers: ATCTGACATCTGGAAGCAGGAT (spanning the *GRN* exon 4–5 junction).

A copy number standard curve was prepared by spiking a serial dilution of the *Tie2-Grn* transgene construct plasmid DNA into 10 ng of WT mouse genomic DNA (gDNA). First, we calculated how much plasmid DNA should be spiked into 10 ng of gDNA in order to reflect one copy of the transgene. In this instance, a single copy is equivalent to 0.013594 pg of *Tie2-Grn* plasmid DNA spiked in 10 ng of gDNA. The amplification efficiency was 1.86, r = 1, error = 0.11, indicating the qPCR condition was optimized and this was a good standard curve. An external standard curve was created and re-used for copy number estimation. Measurements were performed in triplicate.

### Determination of *Tie2-Grn* Transgene mRNA Expression in Mouse Tissue by Real Time RT-PCR

Total RNAs were extracted from mouse tissues (brain, lung, liver, kidney, and skin) using Qiagen RNeasy Micro Kit according to the manufacturer’s instructions. Real-time quantitative RT-PCR analysis for *Tie2-Grn* mRNA and endogenous *GRN* mRNA expression was performed using a LightCycler 2.0 System (Roche).

To selectively detect *Tie2-Grn* mRNA expression and exclude endogenous *GRN* expression, we used the following primers:

Forward primer: TTGCGAAAGAAGATTCCTCGCTGG (close to 3′-UTR region, exon 13);

Reverse primer: ACCTCTACAGATGTGATATGGCTG (polyA region on the transgene construct).

To specifically detect endogenous *GRN* mRNA expression, we designed a primer set close to 3′-UTR, but after the *GRN* stop codon (in the end of exon 13).

Forward primer: TCTTCCGGTTTCTGTGGACCTTGT;

reverse primer: TTATTGGAGCAACACACGCACACG.

Mouse β-actin was used as housekeeping control using the same primers as above.

All the PCR primers were obtained from AlphaDNA (Montreal, Quebec).

Amplification reactions were performed using the QuantiTect SYBR Green PCR kit. (Roche) The thermal cycling conditions comprised an initial denaturation step at 95°C for 15 min followed by 40 cycles amplification at 95°C for 10 s, 66°C for 5 s, and 72°C for 10 s to amplify *Tie2-Grn* transgene, endogenous *Grn* and *β-Actin*. The PCR products were subsequently melted at 60°C for 30 s. The melting curve analysis shows a single peak with no primer-dimers at the described PCR working conditions for all the genes amplified. PCR without reverse-transcribed cDNA were used as negative controls.

### Genotyping

Embryo yolk sacs, fetal, neonatal or adult tail snips were incubated in lysis buffer (10 mM Tris, pH 8.0, 100 mM NaCl, 10 mM EDTA, 0.5% SDS) with proteinase K (0.4 mg/mL) at 55°C overnight with gentle shaking at 80 rpm. Genomic DNA was extracted and purified using phenol chloroform. Proteinase K and phenol chloroform were purchased from Bioshop, Canada.

### ELISA

Amniotic fluid was collected from amniotic cavities within the uterus (E14.5–E16.5) and snapped frozen in liquid nitrogen. Blood was collected from the heart of the adult mice (2–3 months old) immediately after euthanasia, and allowed to clot at room temperature for 2 hours, and spun at 2000 rpm for 20 minutes. The serum from the blood samples were also snapped frozen in liquid nitrogen. The frozen amniotic fluid and serum samples were thawed on ice and subjected to respective ELISA. The progranulin ELISA kit was purchased from Cayman Chemical Company and the VEGF ELISA kit was purchased from R&D Systems.

### Immunohistochemistry

Mouse embryos or tissues were collected for histological assessment. Samples were fixed in 10% neutral buffered formalin (Fisher) and embedded in paraffin. Sections were deparaffinized in Citrosolv (Fisher) and rehydrated in a graded series of ethanol. Antigen retrieval was performed by boiling the slides in a 10 mM sodium citrate solution for 20 min in a microwave oven. The sections were incubated with Dako Dual Endogenous Enzyme Block (DakoCytomation) for an hour at room temperature using the LSAB+ System-HRP (DakoCytomation) as per the manufacturer’s recommended protocol. Positive reactions were detected using Dako Liquid DAB+ Substrate Chromogen System (DakoCytomation). The primary antibodies employed were diluted in Dako Antibody Diluent (DakoCytomation). The anti-mouse progranulin antibody (dilution 1∶70) was purchased from R&D systems, the anti-laminin antibody (dilution 1∶50) was purchased from Abcam, the anti-smooth muscle α-actin antibody (dilution 1∶100) and the anti-desmin (dilution 1∶100) were purchased from Dako; the anti-PECAM1 antibody (dilution 1∶25) was purchased from Santa Cruz and the VEGFR2 antibody (dilution 1∶600) was purchased from Cell Signaling.

## Supporting Information

Figure S1 **Total red blood cell counts from** ***Tie2-Grn*** **positive and negative newborn mice.** Independent samples T-test revealed insignificant differences (*p* = 0.054) in red blood cell number between the *Tie2-Grn* positive and the negative mice. Error bars denote 95% C.I. Within the *Tie2-Grn* positive population, the total red blood cell count was not correlated with pallor, *p* = 0.821 (not shown).
(TIF)

Figure S2 **Mean weight of** ***Tie2-Grn*** **positive and negative mice.** Data taken from 3-week-old mice (weaning age). Independent samples T-test was performed on individual lines, *p* = 0.943 (GrnLo), *p* = 0.940 (GrnMid) and *p*<0.001 (GrnHi). Tie2*-Grn* positive mice that survived birth had similar average weight as *Tie2-Grn* negative littermates, except in the GrnHi line where *Tie2-Grn* negative mice were heavier than their *Tie2-Grn* positive counterparts. *Tie2-Grn* positive mice from GrnHi were of similar average weight as *Tie2-Grn* positive and negative mice from the GrnLo and GrnMid lines. Error bars indicate +/− S.E. Asterisks indicate statistical significance in S2; significance of <0.001 is ***, <0.01 is ** and <0.05 is *.
(TIF)

Figure S3 Lung section of a *Tie2-Grn* negative adult showing (A) absence of endothelial progranulin protein expression and (B) positive aSMA protein expression in the same blood vessel (serial sections), as indicated by the arrowheads. Lung section of a *Tie2-Grn* positive adult showing (C) positive endothelial progranulin protein expression and (D) positive aSMA protein expression in the same blood vessel (serial sections), as indicated by the arrowheads. (E) Weak progranulin expression in a neighboring vessel of the same lung section as C and D. Scale bars denote 20 µm. Images were obtained from the GrnHi line. GrnHi adults photographed were about 3 months old.
(TIF)

Figure S4 **Fetal placenta at E15.5 from** ***Tie2-Grn*** **positive and negative mice.** The endothelial walls of blood vessels in the fetal placenta stain for progranulin in the *Tie2-Grn* negative mice (A) and *Tie2-Grn* positive (B) mice (arrowheads). H&E stains of the placenta indicate that placental structure is apparently unaffected by the expression of *Tie2-Grn* transgene comparing the *Tie2-Grn* negative (C) and positive (D) mice. However, the *Tie2-Grn* positive placenta showed larger, and more frequent syncytial knots than their *Tie2-Grn* negative counterparts (see insert boxes in C and D). Scale bars denote 20 µm.
(TIF)

Table S1 Statistical analysis of the mean weights (g) of mice at 3 weeks. Independent variables, gender, genotype and litter size were tested using a Univariate General Linear Model. No statistical significance was attained between mean weight at birth and genotype (*p = *0.144) or gender (*p = *0.627), but it was significant for litter size *(p*<0.001).
(DOC)

Video S1 **Pale colored pups were born alive.** The video was taken within hours of birth.
(AVI)

Video S2 **Some pale colored neonates displayed apparent respiratory distress.** The video was taken shortly after birth.
(AVI)

## References

[1] Bao P, Kodra A, Tomic-Canic M, Golinko MS, Ehrlich HP, et al (2009) The role of vascular endothelial growth factor in wound healing. Journal of Surgical Research 153: 347–358. 19027922 10.1016/j.jss.2008.04.023 PMC2728016

[2] Bishop A (2008) Role of oxygen in wound healing. Journal of Wound Care 17: 399–402. 18833899 10.12968/jowc.2008.17.9.30937

[3] Demir R, Yaba A, Huppertz B (2010) Vasculogenesis and angiogenesis in the endometrium during menstrual cycle and implantation. Acta Histochemica 112: 203–214. 19481785 10.1016/j.acthis.2009.04.004

[4] Andres AC, Djonov V (2010) The mammary gland vasculature revisited. Journal of Mammary Gland Biology and Neoplasia 15: 319–328. 20706777 10.1007/s10911-010-9186-9

[5] Cao Y (2007) Angiogenesis modulates adipogenesis and obesity. Journal of Clinical Investigation 117: 2362–2368. 17786229 10.1172/JCI32239 PMC1963348

[6] Lijnen HR (2008) Angiogenesis and obesity. Cardiovascular Research 78: 286–293. 18006485 10.1093/cvr/cvm007

[7] Papetti M, Herman IM (2002) Mechanisms of normal and tumor-derived angiogenesis. Am J Physiol Cell Physiol 282: C947–970. 11940508 10.1152/ajpcell.00389.2001

[8] Praidou A, Androudi S, Brazitikos P, Karakiulakis G, Papakonstantinou E, et al (2010) Angiogenic growth factors and their inhibitors in diabetic retinopathy. Curr Diabetes Rev 6: 304–312. 20594164 10.2174/157339910793360815

[9] Nakagawa T, Kosugi T, Haneda M, Rivard CJ, Long DA (2009) Abnormal angiogenesis in diabetic nephropathy. Diabetes 58: 1471–1478. 19564458 10.2337/db09-0119 PMC2699857

[10] Sullivan DC, Bicknell R (2003) New molecular pathways in angiogenesis. British Journal of Cancer 89: 228–231. 12865906 10.1038/sj.bjc.6601107 PMC2394258

[11] He ZH, Ong CHP, Halper J, Bateman A (2003) Progranulin is a mediator of the wound response. Nature Medicine 9: 225–229. 10.1038/nm816 12524533

[12] Bateman A, Bennett HP (2009) The granulin gene family: from cancer to dementia. Bioessays 31: 1245–1254. 19795409 10.1002/bies.200900086

[13] Ong CHP, Bateman A (2003) Progranulin (Granulin-epithelin precursor, PC-cell derived growth factor, Acrogranin) in proliferation and tumorigenesis. Histology and Histopathology 18: 1275–1288. 12973694 10.14670/HH-18.1275

[14] Toh H, Chitramuthu BP, Bennett HP, Bateman A (2011) Structure, function, and mechanism of progranulin; the brain and beyond. Journal of Molecular Neuroscience 45: 538–548. 21691802 10.1007/s12031-011-9569-4

[15] Elkabets M, Gifford AM, Scheel C, Nilsson B, Reinhardt F, et al (2011) Human tumors instigate granulin-expressing hematopoietic cells that promote malignancy by activating stromal fibroblasts in mice. Journal of Clinical Investigation 121: 784–799. 21266779 10.1172/JCI43757 PMC3026724

[16] Baker M, Mackenzie IR, Pickering-Brown SM, Gass J, Rademakers R, et al (2006) Mutations in progranulin cause tau-negative frontotemporal dementia linked to chromosome 17. Nature 442: 916–919. 16862116 10.1038/nature05016

[17] Cruts M, Gijselinck I, van der Zee J, Engelborghs S, Wils H, et al (2006) Null mutations in progranulin cause ubiquitin-positive frontotemporal dementia linked to chromosome 17q21. Nature 442: 920–924. 16862115 10.1038/nature05017

[18] Zhu J, Nathan C, Jin W, Sim D, Ashcroft GS, et al (2002) Conversion of proepithelin to epithelins: roles of SLPI and elastase in host defense and wound repair. Cell 111: 867–878. 12526812 10.1016/s0092-8674(02)01141-8

[19] Yin FF, Banerjee R, Thomas B, Zhou P, Qian LP, et al (2010) Exaggerated inflammation, impaired host defense, and neuropathology in progranulin-deficient mice. Journal of Experimental Medicine 207: 117–128. 20026663 10.1084/jem.20091568 PMC2812536

[20] Kessenbrock K, Frohlich L, Sixt M, Lammermann T, Pfister H, et al (2008) Proteinase 3 and neutrophil elastase enhance inflammation in mice by inactivating antiinflammatory progranulin. J Clin Invest 118: 2438–2447. 18568075 10.1172/JCI34694 PMC2430496

[21] Tang W, Lu Y, Tian QY, Zhang Y, Guo FJ, et al (2011) The growth factor progranulin binds to TNF receptors and is therapeutic against inflammatory arthritis in mice. Science 332: 478–484. 21393509 10.1126/science.1199214 PMC3104397

[22] Park B, Buti L, Lee S, Matsuwaki T, Spooner E, et al (2011) Granulin is a soluble cofactor for toll-like receptor 9 signaling. Immunity 34: 505–513. 21497117 10.1016/j.immuni.2011.01.018

[23] Daniel R, He ZH, Carmichael KP, Halper J, Bateman A (2000) Cellular localization of gene expression for progranulin. Journal of Histochemistry & Cytochemistry 48: 999–1009. 10858277 10.1177/002215540004800713

[24] Desmarais JA, Cao MJ, Bateman A, Murphy BD (2008) Spatiotemporal expression pattern of progranulin in embryo implantation and placenta formation suggests a role in cell proliferation, remodeling, and angiogenesis. Reproduction 136: 247–257. 18469036 10.1530/REP-08-0044

[25] Davidson B, Alejandro E, Florenes VA, Goderstad JM, Risberg B, et al (2004) Granulin-epithelin precursor is a novel prognostic marker in epithelial ovarian carcinoma. Cancer 100: 2139–2147. 15139056 10.1002/cncr.20219

[26] Gonzalez EM, Mongiat M, Slater SJ, Baffa R, Iozzo RV (2003) A novel interaction between perlecan protein core and progranulin: potential effects on tumor growth. Journal of Biological Chemistry 278: 38113–38116. 12900424 10.1074/jbc.C300310200

[27] Li LQ, Min LS, Jiang Q, Ping JL, Li J, Dai LC (2012) Progranulin expression in breast cancer with different intrinsic subtypes. Pathol Res Pract. Apr 15 208(4): 210–6. 10.1016/j.prp.2012.02.001 22397762

[28] Chen XY, Li JS, Liang QP, He DZ, Zhao J (2008) Expression of PC cell-derived growth factor and vascular endothelial growth factor in esophageal squamous cell carcinoma and their clinicopathologic significance. Chinese Medical Journal 121: 881–886. 18706200

[29] Wang M, Li G, Yin J, Lin T, Zhang J (2012) Progranulin overexpression predicts overall survival in patients with glioblastoma. Medical Oncology. 10.1007/s12032-011-0131-6 22161130

[30] Tangkeangsirisin W, Serrero G (2004) PC cell-derived growth factor (PCDGF/GP88, progranulin) stimulates migration, invasiveness and VEGF expression in breast cancer cells. Carcinogenesis 25: 1587–1592. 15117809 10.1093/carcin/bgh171

[31] Ho JC, Ip YC, Cheung ST, Lee YT, Chan KF, et al (2008) Granulin-epithelin precursor as a therapeutic target for hepatocellular carcinoma. Hepatology 47: 1524–1532. 18393387 10.1002/hep.22191

[32] Ponce ML (2001) Angiogenesis Protocols; Murray J, editor. Totowa, NJ: Humana Press.

[33] Schnurch H, Risau W (1993) Expression of tie-2, a member of a novel family of receptor tyrosine kinases, in the endothelial cell lineage. Development 119: 957–968. 8187650 10.1242/dev.119.3.957

[34] Schlaeger TM, Bartunkova S, Lawitts JA, Teichmann G, Risau W, et al (1997) Uniform vascular-endothelial-cell-specific gene expression in both embryonic and adult transgenic mice. Proceedings of the National Academy of Sciences of the United States of America 94: 3058–3063. 9096345 10.1073/pnas.94.7.3058 PMC20321

[35] Evans V, Hatzopoulos A, Aird WC, Rayburn HB, Rosenberg RD, et al (2000) Targeting the Hprt locus in mice reveals differential regulation of Tie2 gene expression in the endothelium. Physiol Genomics 2: 67–75. 11015584 10.1152/physiolgenomics.2000.2.2.67

[36] Pereira R, Halford K, Sokolov BP, Khillan JS, Prockop DJ (1994) Phenotypic variability and incomplete penetrance of spontaneous fractures in an inbred strain of transgenic mice expressing a mutated collagen gene (COL1A1). Journal of Clinical Investigation 93: 1765–1769. 8163675 10.1172/JCI117161 PMC294239

[37] Miko I (2008) Phenotype Variability: Penetrance and Expressivity. Nature Education 1.

[38] Drake CJ, Fleming PA (2000) Vasculogenesis in the day 6.5 to 9.5 mouse embryo. Blood 95: 1671–1679. 10688823

[39] Person AD, Klewer SE, Runyan RB (2005) Cell biology of cardiac cushion development. International Review of Cytology 243: 287–335. 15797462 10.1016/S0074-7696(05)43005-3

[40] Schroeder JA, Jackson LF, Lee DC, Camenisch TD (2003) Form and function of developing heart valves: coordination by extracellular matrix and growth factor signaling. J Mol Med (Berl) 81: 392–403. 12827270 10.1007/s00109-003-0456-5

[41] Mongiat M, Fu J, Oldershaw R, Greenhalgh R, Gown AM, et al (2003) Perlecan protein core interacts with extracellular matrix protein 1 (ECM1), a glycoprotein involved in bone formation and angiogenesis. Journal of Biological Chemistry 278: 17491–17499. 12604605 10.1074/jbc.M210529200

[42] Handler M, Yurchenco PD, Iozzo RV (1997) Developmental expression of perlecan during murine embryogenesis. Developmental Dynamics 210: 130–145. 9337134 10.1002/(SICI)1097-0177(199710)210:2<130::AID-AJA6>3.0.CO;2-H

[43] Segev A, Nili N, Strauss BH (2004) The role of perlecan in arterial injury and angiogenesis. Cardiovascular Research 63: 603–610. 15306215 10.1016/j.cardiores.2004.03.028

[44] Hellstrom M, Kalen M, Lindahl P, Abramsson A, Betsholtz C (1999) Role of PDGF-B and PDGFR-beta in recruitment of vascular smooth muscle cells and pericytes during embryonic blood vessel formation in the mouse. Development 126: 3047–3055. 10375497 10.1242/dev.126.14.3047

[45] Hellstrom M, Gerhardt H, Kalen M, Li X, Eriksson U, et al (2001) Lack of pericytes leads to endothelial hyperplasia and abnormal vascular morphogenesis. Journal of Cell Biology 153: 543–553. 11331305 10.1083/jcb.153.3.543 PMC2190573

[46] Zeng X, Wert SE, Federici R, Peters KG, Whitsett JA (1998) VEGF enhances pulmonary vasculogenesis and disrupts lung morphogenesis in vivo. Developmental Dynamics 211: 215–227. 9520109 10.1002/(SICI)1097-0177(199803)211:3<215::AID-AJA3>3.0.CO;2-K

[47] Heazell AE, Moll SJ, Jones CJ, Baker PN, Crocker IP (2007) Formation of syncytial knots is increased by hyperoxia, hypoxia and reactive oxygen species. Placenta 28 Suppl A: S33–40. 10.1016/j.placenta.2006.10.007 17140657

[48] Trampont P, Roudier M, Andrea AM, Nomal N, Mignot TM, et al (2004) The placental-umbilical unit in sickle cell disease pregnancy: a model for studying in vivo functional adjustments to hypoxia in humans. Human Pathology 35: 1353–1359. 15668892 10.1016/j.humpath.2004.07.003

[49] Khalid ME, Ali ME, Ali KZ (1997) Full-term birth weight and placental morphology at high and low altitude. International Journal of Gynaecology and Obstetrics 57: 259–265. 9215488 10.1016/s0020-7292(97)00067-2

[50] Shalaby F, Rossant J, Yamaguchi TP, Gertsenstein M, Wu XF, et al (1995) Failure of blood-island formation and vasculogenesis in Flk-1-deficient mice. Nature 376: 62–6. 7596435 10.1038/376062a0

[51] Carmeliet P, Ferreira V, Breier G, Pollefeyt S, Kieckens L, et al (1996) Abnormal blood vessel development and lethality in embryos lacking a single VEGF allele. Nature 380: 435–439. 8602241 10.1038/380435a0

[52] Ferrara N, Carver-Moore K, Chen H, Dowd M, Lu L, et al (1996) Heterozygous embryonic lethality induced by targeted inactivation of the VEGF gene. Nature 380: 439–442. 8602242 10.1038/380439a0

[53] Suri C, Jones PF, Patan S, Bartunkova S, Maisonpierre PC, et al (1996) Requisite role of angiopoietin-1, a ligand for the TIE2 receptor, during embryonic angiogenesis. Cell 87: 1171–80. 8980224 10.1016/s0092-8674(00)81813-9

[54] Abramsson A, Berlin O, Papayan H, Paulin D, Shani M, Betsholtz C (2002) Analysis of mural cell recruitment to tumor vessels. Circulation 105: 112–7. 11772885 10.1161/hc0102.101437

[55] Silver LM (1995) Mouse Genetics Concepts and Applications: Oxford University Press.

