# Supplementary figures and images for: Expression of the Growth Factor Progranulin in Endothelial Cells Influences Growth and Development of Blood Vessels: A Novel Mouse Model
Source: PLoS One. 2013 May 31;8(5):e64989. doi: 10.1371/journal.pone.0064989 (PMC3669103; doi:10.1371/journal.pone.0064989)

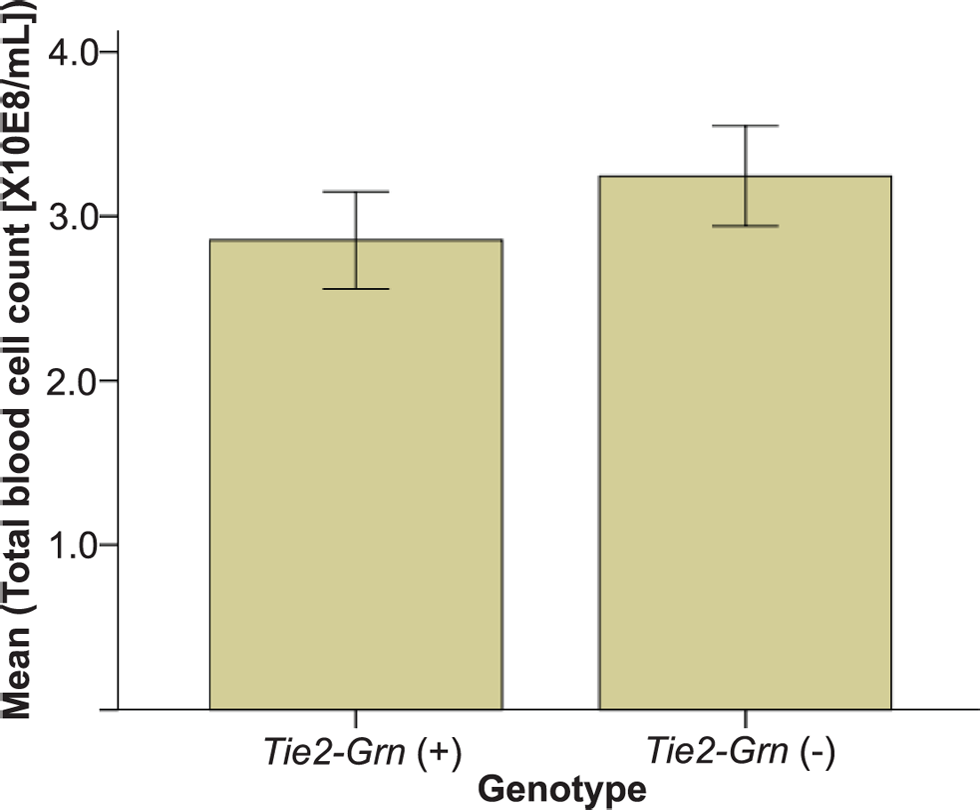

Supplement: Figure S1 — Total red blood cell counts from Tie2-Grn positive and negative newborn mice. Independent samples T-test revealed insignificant differences (p = 0.054) in red blood cell number between the Tie2-Grn positive and the negative mice. Error bars denote 95% C.I. Within the Tie2-Grn positive population, the total red blood cell count was not correlated with pallor, p = 0.821 (not shown). (TIF) [file pone.0064989.s001.tif]

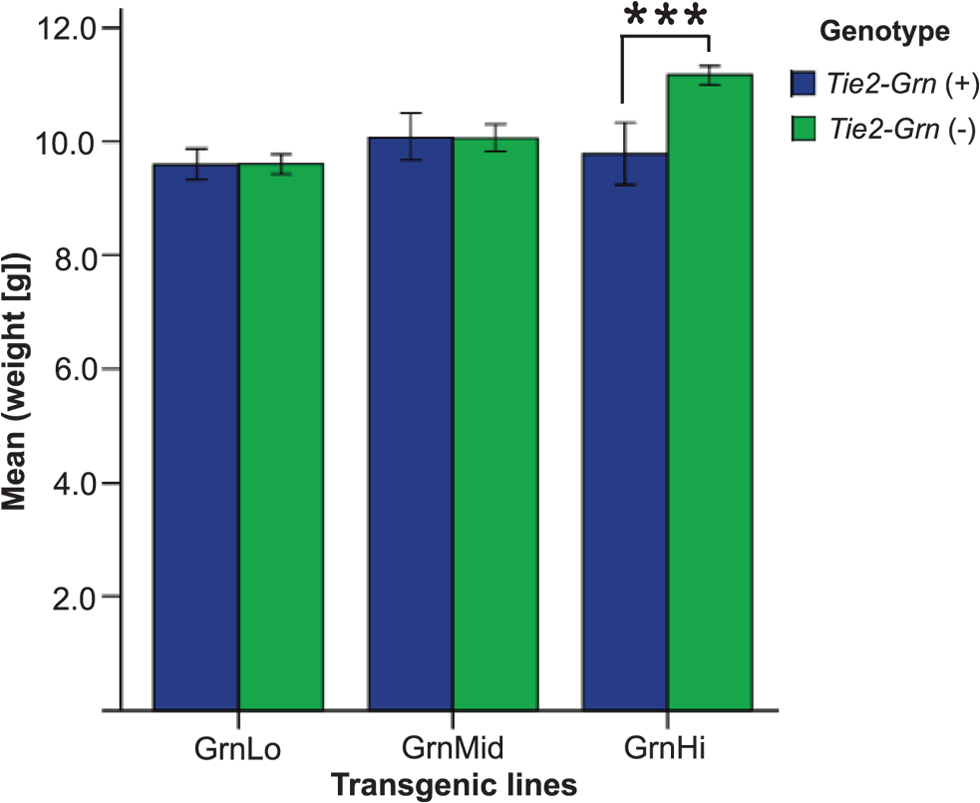

Supplement: Figure S2 — Mean weight of Tie2-Grn positive and negative mice. Data taken from 3-week-old mice (weaning age). Independent samples T-test was performed on individual lines, p = 0.943 (GrnLo), p = 0.940 (GrnMid) and p<0.001 (GrnHi). Tie2-Grn positive mice that survived birth had similar average weight as Tie2-Grn negative littermates, except in the GrnHi line where Tie2-Grn negative mice were heavier than their Tie2-Grn positive counterparts. Tie2-Grn positive mice from GrnHi were of similar average weight as Tie2-Grn positive and negative mice from the GrnLo and GrnMid lines. Error bars indicate +/− S.E. Asterisks indicate statistical significance in S2; significance of <0.001 is ***, <0.01 is ** and <0.05 is *. (TIF) [file pone.0064989.s002.tif]

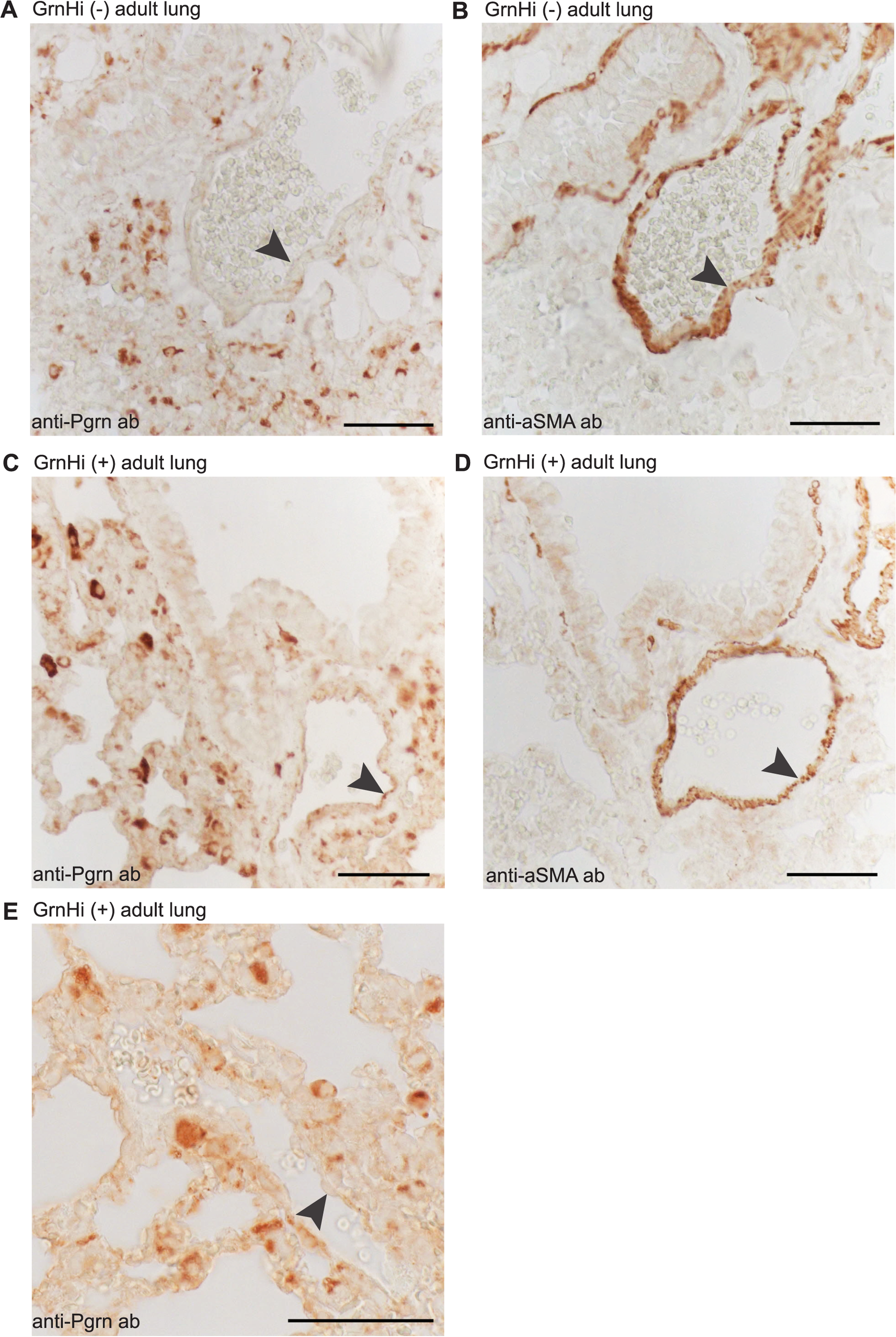

Supplement: Figure S3 — Lung section of a Tie2-Grn negative adult showing (A) absence of endothelial progranulin protein expression and (B) positive aSMA protein expression in the same blood vessel (serial sections), as indicated by the arrowheads. Lung section of a Tie2-Grn positive adult showing (C) positive endothelial progranulin protein expression and (D) positive aSMA protein expression in the same blood vessel (serial sections), as indicated by the arrowheads. (E) Weak progranulin expression in a neighboring vessel of the same lung section as C and D. Scale bars denote 20 µm. Images were obtained from the GrnHi line. GrnHi adults photographed were about 3 months old. (TIF) [file pone.0064989.s003.tif]

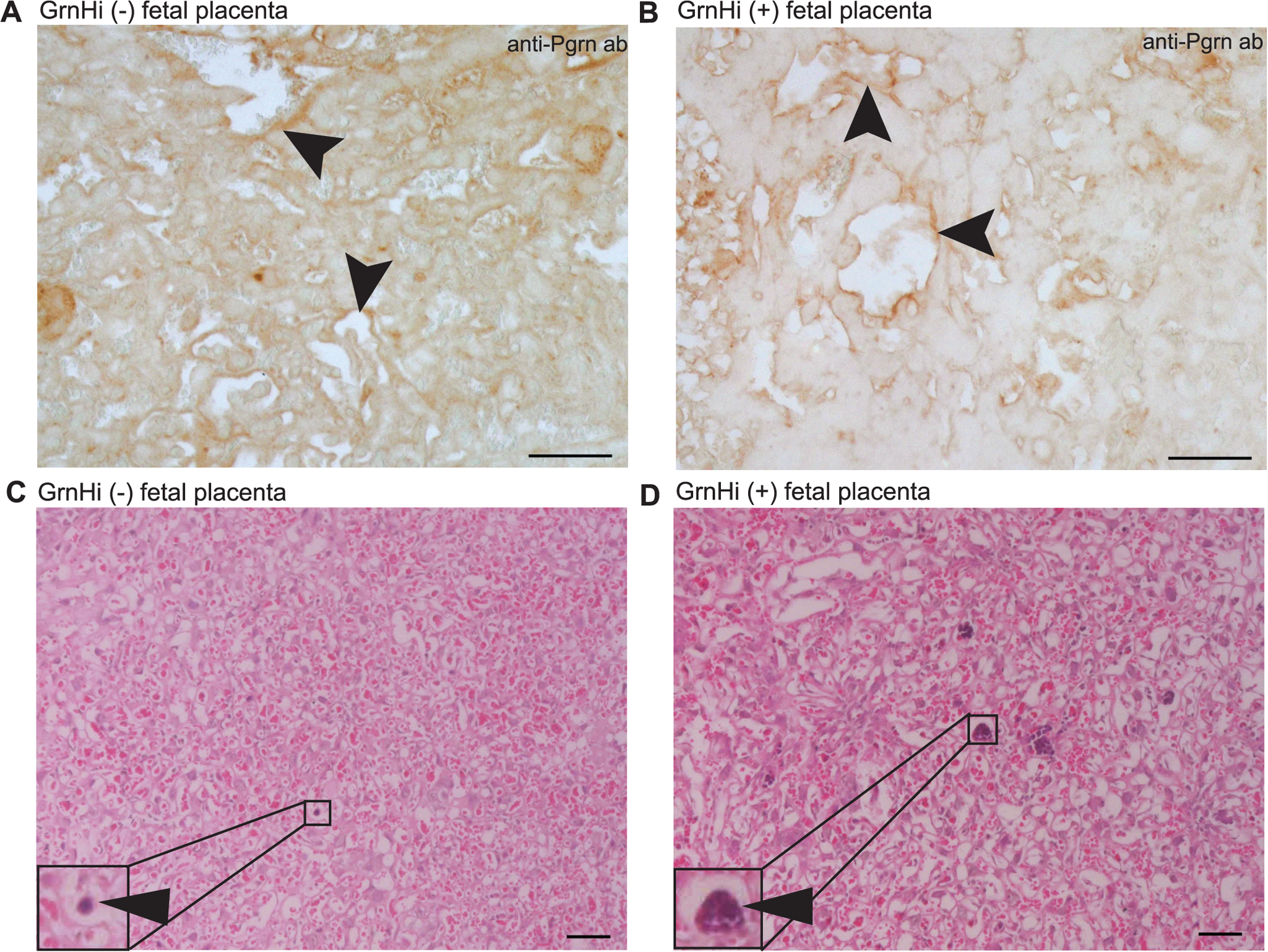

Supplement: Figure S4 — Fetal placenta at E15.5 from Tie2-Grn positive and negative mice. The endothelial walls of blood vessels in the fetal placenta stain for progranulin in the Tie2-Grn negative mice (A) and Tie2-Grn positive (B) mice (arrowheads). H&E stains of the placenta indicate that placental structure is apparently unaffected by the expression of Tie2-Grn transgene comparing the Tie2-Grn negative (C) and positive (D) mice. However, the Tie2-Grn positive placenta showed larger, and more frequent syncytial knots than their Tie2-Grn negative counterparts (see insert boxes in C and D). Scale bars denote 20 µm. (TIF) [file pone.0064989.s004.tif]
