# Supplementary material for: Expression of the Growth Factor Progranulin in Endothelial Cells Influences Growth and Development of Blood Vessels: A Novel Mouse Model
Source: PLoS One. 2013 May 31;8(5):e64989. doi: 10.1371/journal.pone.0064989 (PMC3669103; doi:10.1371/journal.pone.0064989)
Supplement: Table S1 — Statistical analysis of the mean weights (g) of mice at 3 weeks. Independent variables, gender, genotype and litter size were tested using a Univariate General Linear Model. No statistical significance was attained between mean weight at birth and genotype (p = 0.144) or gender (p = 0.627), but it was significant for litter size (p<0.001). (DOC) [file pone.0064989.s005.doc]

Univariate GLM - Dependent Variable: weight of mice at 3 weeks

| **Source** | **Type III Sum of Squares** | **Degrees of Freedom** | **Mean Square** | **F-value** | **Significance** |
| --- | --- | --- | --- | --- | --- |
| Gender | 0.636 | 1 | 0.636 | 0.237 | 0.627 |
| Genotype | 5.748 | 1 | 5.748 | 2.137 | 0.144 |
| Litter size | 549.536 | 10 | 54.954 | 20.435 | 0.000 |
